# Rabies, Still Neglected after 125 Years of Vaccination

**DOI:** 10.1371/journal.pntd.0000839

**Published:** 2010-11-30

**Authors:** Hervé Bourhy, Alice Dautry-Varsat, Peter J. Hotez, Jérôme Salomon

**Affiliations:** 1 Institut Pasteur, Paris, France; 2 Sabin Vaccine Institute, Washington, D. C., United States of America; 3 Department of Microbiology, Immunology, and Tropical Medicine, George Washington University, Washington, D.C., United States of America

Rabies is a viral zoonotic infection of the central nervous system caused by a lyssavirus. The disease is fatal without proper post exposure prophylaxis (PEP). In July 1885, Louis Pasteur obtained his first success against rabies by vaccinating Joseph Meister, a 9-year-old boy presenting with multiple deep bite wounds [1]. After more than 700 successful inoculations, Pasteur launched an international subscription and opened the world's first research institute dedicated to the prevention of rabies and other infectious diseases. The Institut Pasteur was born. Due to substantial advances during the 20th century, safe and effective human and animal vaccines based on tissue culture methodologies are available today. Today, 125 years later, Institut Pasteur is at the core of an international network of 30 institutes. Through its numerous institutions established in enzootic areas, the worldwide contribution of the Institut Pasteur International Network to rabies surveillance and control is still of paramount importance. Each year, the International Network is responsible for PEP administration to more than 180,000 exposed patients, mainly in Southeast Asia and Africa. Scientists and health staff are also involved in various national committees that work on the development and successful implementation of rabies control programs.

Sadly, whereas extensive efforts in developed countries have largely controlled dog (the United States and Europe) and fox (western and central Europe) rabies [2], [3], dog rabies remains enzootic in much of the world, and 15 million people require PEP every year. Rabies is considered one of the most neglected diseases in the world's developing countries with the greatest burden in poor rural communities, and disproportionately in children. According to the World Health Organization (WHO), 30% to 50% of the 55,000 victims estimated each year are individuals under 15 years of age [4]. Over 95% of these human rabies cases are concentrated in Asia (especially in India) and Africa, and 99% of them are transmitted by dogs. There are several reasons for the lack of accurate data, including weak or non-existent rabies surveillance systems; under-reporting of cases by local communities and central authorities; unreliable diagnosis of cases, which is generally based on clinical criteria rather than laboratory confirmation; and inadequate legislation for compulsory notification of cases. The absence of accurate data on disease burden, upon which regional and national priorities for research and control are based, leads to a vicious cycle of indifference and neglect [5].

The question now is why, despite the availability of safe and effective human vaccines, human rabies deaths continue to escalate in many parts of the world. There are several general and specific explanations for the continued burden of dog rabies [6]–[8].

In general, there is a lack of awareness amongst policy-makers of the rabies burden and impacts and the need for prioritizing resources towards its control. Further, despite the widely advocated need for intersectoral collaboration between government ministries, the recognition of roles and responsibilities amongst agencies as well as integration of budgets across ministries still poses considerable challenges.

Mass vaccination of dogs is the most cost-effective way to achieve a significant and lasting reduction in the number of human deaths from rabies [9]. However, such prevention efforts are often not prioritized by national governments in even the most endemic countries. Among the reasons for the low priority afforded rabies are, as for many neglected tropical diseases, its predominance in remote rural areas and its disproportionate impact on people living in extreme poverty. Therefore, lack of resources, gaps in epidemiological knowledge (e.g., level of vaccination coverage needed in any given setting), lack of in-country expertise on implementation of canine rabies control strategies, and sustainability issues impair the success of such programs.

Additionally, in most developing countries (with some notable exceptions), there is a lack of awareness among the population, including medical practitioners and health authorities, about the widespread extent of the disease and the risk of transmission from dogs to human populations [6]–[8]. Additionally, the infrastructure for the management of rabies exposure is scarce. Many people who are exposed to rabies do not seek PEP, because they are not aware of the risk of contracting this disease or because they live in rural areas too far away from rabies prevention centers, which are generally located in big cities. In some cases, the world's poorest people simply cannot afford the cost of PEP.

A range of key scientific questions also remains unsolved, and there is an urgency to promote research and development on the fundamental aspects of rabies molecular pathobiology, improved control tools, and operational research. There is also a need for multidisciplinary integrative approaches that rely on the following elements.

1. It is essential to find ways to improve epidemiological surveillance using diagnostic approaches to rabies based on validated protocols and specimens and evaluated under field conditions [10]–[12]. Epidemiological models are also needed to better estimate the incidence of rabies [4], [13], [14]. Recent research on this topic published in this journal yielded rabies incidence for some countries that could be as high as 15 times that of the official reports [15].
2. Information concerning the epidemiology and population dynamics of rabies in its natural animal host population, particularly dogs (involved in more than 99% of human fatalities), is lacking. Understanding the ecological patterns, frequency, and extent of movement of virally infected animals is of paramount importance in predicting the spread of zoonotic infections [16], [17], and hence is essential for their ultimate control. Also, work on dog population studies and their accessibility to vaccination is still needed. Some recent epidemiological studies have made advances in this area [14], [18]–[21]. Further, extensive genomic and evolutionary analyses establishing the diversity of lyssavirus species and variants will help us to better understand the determinants of rabies spread. The analysis of integrated phylogeographic data can now transform viral genetic data into a powerful asset for characterizing, predicting, and potentially controlling the spatial spread of rabies and, more largely, of pathogens [22]–[24]. Understanding the conditions under which the containment of dog rabies can reliably be achieved will facilitate the long-term goal of its elimination.
3. Cheaper and innovative therapeutic approaches are urgently required. Currently, the recommended treatment for individuals exposed to rabies virus is the combined administration of rabies vaccine and immunoglobulins [25], [26]. Both of these biologics remain relatively expensive for a significant portion of the target population. The average cost per rabies vaccination, which is the sum of direct and indirect costs with vaccine application alone, is US$45 in Africa and Asia (whereas the average salary is often less than US$2 per day in most endemic countries). When rabies immunoglobulins are also provided, the cost is around US$100 per patient [4]. Therefore, it is necessary to search for ways and means to decrease the cost of rabies biologicals. Recently, efforts to develop new generation biologics comprised of mixtures of human anti-rabies monoclonal antibodies have yielded promising results [27]. Antiviral therapy to treat rabid patients is also lacking. Exploring innovative therapeutic approaches based on blocking interactions among viral proteins will probably lead to the discovery and development of new small molecule compounds [28]–[30]. Although tools for successful dog rabies control are available, and other factors are responsible for the failure to bring dog rabies under control in most of the developing world [20], present efforts in translational and operational research on innovative veterinary vaccines and modes of delivery will have to be continued in order to provide better and easier control of the animal reservoir in tropical and resource-poor conditions [31]–[34].
4. A comprehensive understanding of the pathobiology of rabies viruses is lacking. Many studies are now addressing the nature of the relationships among lyssaviruses and their hosts [35]–[37], but the respective role of different viral proteins and how they affect the host cellular machinery remains largely a mystery [38]–[40]. An analysis comparing the pathogenic processes in different lyssaviruses found in nature with different human susceptibilities could be central to answering this question.

In conclusion, we strongly recommend the establishment of a comprehensive national rabies control program in each of the world's enzootic countries to ensure continued political commitment and active community participation. Also, once established, international support will have to be provided to implement and sustain rabies control activities. The universal establishment of national programs could also help to ensure that only WHO-recommended rabies vaccines are administered and made available to local populations, and that dog rabies transmission is controlled and ultimately eliminated or eradicated. Since 2007, World Rabies Day (http://www.worldrabiesday.org/) has been held every September 28th (the day of Louis Pasteur's death). This commemoration is an invaluable advocacy tool for increasing awareness in local populations, informing them about the effects of the disease in humans and animals, promoting existing preventive measures, and ultimately eradicating human rabies by focusing on the control of animal reservoirs.

The legacy of Louis Pasteur reminds us that despite rapidly shifting priorities due to periodic recognition of emerging diseases, none of these other conditions exceeds the case fatality rate of rabies. Substantive coordinated efforts towards building and strengthening medical, veterinarian, scientific, and research capacities towards rabies control must be made. This comprehensive and intersectoral approach requires an increasing involvement of philanthropic organizations, government funding agencies, and public–private partnerships to promote political awareness and expand funding opportunities. Through its numerous research, development, and public health activities, the Institut Pasteur International Network continues its 125-year-long commitment to fight this deadly disease. Together with local scientific institutes and vaccine and other biologics manufacturers, the WHO and its regional offices, the World Organization for Animal Health, the Food and Agriculture Organization, and the affected communities themselves, the Institut Pasteur wishes to commemorate this anniversary by supporting coalitions of world partners that have come together to improve rabies prevention capacities in developing countries, and to eliminate this ancient scourge. The members of the Institut Pasteur International Network, together with national reference centers and WHO Collaborating Centers, are working actively to develop specific multidisciplinary strategies that promote many of the existing coalitions already committed to rabies (e.g., Alliance for Rabies Control with initiatives including Partners for Rabies Prevention, World Rabies Day, and the Blueprint for Rabies Prevention and Control). When implemented globally, these strategies will ultimately improve our understanding of the distribution of the disease, the resulting immunological consequences, and the development of new diagnostic techniques, drugs, and vaccines.
